# Comparison of the Clinical Outcomes between Combined Femtosecond Laser-Assisted In Situ Keratomileusis and Corneal Cross Linking versus Combined Small-Incision Lenticule Extraction and Corneal Cross Linking

**DOI:** 10.1155/2022/6994355

**Published:** 2022-01-31

**Authors:** Yu Di, Fei Mo, Ying Li

**Affiliations:** Department of Ophthalmology, Peking Union Medical College Hospital, Chinese Academy of Medical Sciences, Beijing 100730, China

## Abstract

**Purpose:**

The purpose of this study was to compare femtosecond laser-assisted in situ keratomileusis with prophylactic cross linking (FS-LASIK Xtra) and small-incision lenticule extraction with prophylactic cross linking (SMILE Xtra) in terms of their postoperative clinical outcomes.

**Methods:**

In this retrospective study, 24 patients (48 eyes) with myopia and myopia astigmatism were recruited from 2017 to 2018. All patients underwent comprehensive ophthalmic examinations preoperatively and follow-up visits at one and three months. Four patients (eight eyes) in each group were followed up for 12 months. The following were assessed at each visit: uncorrected distance visual acuity (UDVA), manifest refractive spherical equivalent (MRSE), keratometry values, biomechanical properties, anterior and posterior curvature, and corneal pachymetry.

**Results:**

The FS-LASIK Xtra and SMILE Xtra groups both included 24 eyes of 12 patients. At 1, 3, and 12 months after surgery, a UDVA of 20/20 or better was achieved for 91.7%, 91.7%, and 87.5% of individuals, respectively, in the FS-LASIK group and 95.8%, 100%, and 100% in the SMILE group, respectively. For 75% of eyes in the FS-LASIK Xtra group and 100% of eyes in the SMILE Xtra group, the achieved MRSE was within ±1.00D of attempted correction. The LASIK Xtra group had a significantly higher mean MRSE than the SMILE Xtra group at 3 and 12 months after surgery (*P*=0.006, 0.01), and the SMILE Xtra group had significantly higher *K*1 and *K*2 values than the FS-LASIK group at one month after surgery (*P*=0.024, 0.023). Corneal thickness decreased significantly at one month postoperatively and started to increase over the next 12 months in both groups (*P*=0.001). The biomechanical properties showed no significant intergroup differences at the 12-month follow-up.

**Conclusions:**

FS-LASIK Xtra and SMILE Xtra are safe and effective in the correction of myopia and myopia astigmatism, and both procedures have the same effect on postoperative corneal morphology and biomechanics.

## 1. Background

Femtosecond laser-assisted in situ keratomileusis (FS-LASIK) is a common refractive surgical procedure. It has excellent refractive correction ability and offers predictable and stable refractive results [1, 2]. However, FS-LASIK involves the creation of a corneal flap, which may weaken the corneal structure and decrease corneal rigidity. This could increase the risk of corneal ectasia in cases of correction of moderate-to-high myopia [3, 4]. An all-in-one femtosecond laser procedure, known as small-incision lenticule extraction (SMILE), does not require the creation of a flap. The intrastromal lenticule is taken out through a small 2–5 mm incision. This reduces the risks associated with flap creation [5, 6]. Shen et al. [7] proposed that ocular response analyzer and dynamic high-speed Scheimpflug imaging studies have shown that SMILE may better preserve corneal biomechanical properties than LASIK. However, ectasia has also been reported following SMILE [8, 9].

Corneal cross linking (CXL) was first introduced by Wollensak et al. as a promising technique to slow or stop the progression of keratoconus [10]. It also has an excellent record in post-LASIK ectasia [11, 12]. CXL uses the photochemical interaction of ultraviolet A radiation (UVA) and riboflavin (vitamin B2) to induce cross linking between corneal stromal macromolecules, resulting in increased biomechanical stiffness and improved resistance to enzymatic digestion [13]. Recently, CXL has been combined with either LASIK or SMILE to reduce the risk of postoperative keratectasia, and these procedures have been termed LASIK Xtra and SMILE Xtra, respectively. Konstantopoulos et al. [14] proposed that LASIK Xtra and SMILE Xtra showed the smallest increase in maximum posterior elevation (MPE), and the former showed the least potential for ectasia.

However, only one study has compared FS-LASIK Xtra with SMILE Xtra and found that the two procedures are comparable in their postoperative outcomes. However, it did not include corneal biomechanical properties [15]. The present study was performed to compare FS-LASIK Xtra with SMILE Xtra in terms of postoperative outcomes, including visual acuity, refractive error, corneal morphology, and biomechanical properties.

## 2. Methods

### 2.1. Patients

This was a retrospective interventional comparative study that included 48 eyes of 24 patients. Data were collected between May 2017 and November 2018. The study was approved by the Institutional Ethics Committee and was conducted in accordance with the tenets of the Declaration of Helsinki. The inclusion criteria included age ≥18 years, preoperative spherical error from −1.00D to −11.00D, refractive cylinder < −5.00D, stable refractive error for more than 1 year, and willingness and ability to comply with postoperative care. Exclusion criteria included a central corneal thickness (CCT) less than 480 *μ*m, predicted postoperative residual stroma bed thickness less than 280 *μ*m, significant ocular diseases, systematic diseases, or medications that could affect wound healing, pregnancy, or breastfeeding.

### 2.2. Ophthalmic Examinations

All patients underwent comprehensive ophthalmic examinations preoperatively and follow-up visits at one and three months. Four patients (eight eyes) in each group were followed up for 12 months. The following were assessed at each visit: uncorrected distance visual acuity (UDVA), corrected distance visual acuity (CDVA), intraocular pressure (IOP), manifest refractive spherical equivalent (MRSE), anterior segment slit-lamp examination, fundus examination, and corneal tomography (TMS; Japan). An ocular response analyzer (ORA; Reichert Corporation, USA) was used to measure the corneal compensated intraocular pressure (IOPcc), Goldmann correlated IOP value (IOPg), corneal resistance factor (CRF), and corneal hysteresis (CH). A Galilei Dual-Scheimpflug analyzer (GAS; Ziemer Group, Switzerland) assessed the SimK values, anterior instantaneous curvature, posterior axial curvature, and corneal pachymetry.

### 2.3. Surgical Technique

All surgeries were performed by the same surgeon (YL) using the same reproducible technique. The SMILE procedure was performed under topical anesthesia using the VisuMax 500 kHz femtosecond laser (Carl Zeiss Meditec, Jena, Germany). The following parameters were used: cap thickness, 110–120 *μ*m; cap diameter, 7.0–7.5 mm; lenticule diameter, 6.0–6.5 mm with a transition zone of 0.1 mm; side cut incision, 2 mm at the 10 o'clock position of the cornea; and cut energy, 135 nJ. After femtolaser application, a blunt spatula was used to loosen the stromal lenticule, and then, it was removed using forceps. Immediately after removal of the lenticule, 0.22% riboflavin (VibeX Xtra, Avedro) was instilled into the intrastromal pocket and allowed to have a soak time of 90 seconds. The riboflavin was then completely washed out from the pocket using balanced saline solution. This was followed by ultraviolet A irradiation using Avedro's (Avedro Inc.) corneal cross-linking system at 30 mW/cm^2^ for 90 seconds, and the total energy was 2.7 J/cm^2^.

FS-LASIK was performed using the VisuMax 500 kHz femtosecond laser (Carl Zeiss Meditec, Jena, Germany) for flap creation and a WaveLight EX500 excimer laser (Alcon Laboratories Inc.) for refractive correction. The following parameters were used: flap thickness, 90 *μ*m or 110 *μ*m; flap diameter, 8.5 mm; hinge position, 12 o'clock of the cornea; and side cut angle, 120 degrees. After excimer laser treatment, 0.22% riboflavin (VibeX Xtra, Avedro) was instilled onto the exposed stromal bed for 90 seconds. The balanced salt solution was then used to flush the remaining riboflavin from the stromal bed. The flap was repositioned followed by ultraviolet A irradiation using Avedro's (Avedro Inc.) corneal cross-linking system at 30 mW/cm^2^ for 90 seconds, and the total energy was 2.7 J/cm^2^.

After ultraviolet A irradiation, the regimens were prescribed for both eyes as follows: 0.5% levofloxacin eye drops (Cravit, Santen), four times a day for two weeks, 0.5% Loteprednol etabonate ophthalmic suspension (Lotemax, Bausch & Lomb Incorporated) in tapering dosages for four weeks (starting with four times per day), and sodium hyaluronate eye drops, four times a day for four weeks.

### 2.4. Statistical Analysis

Statistical analyses were conducted using SPSS statistical software version 23.0 (IBM, Armonk, NY, USA). UDVA and CDVA were converted to the logarithm of the minimum angle of resolution (logMAR) equivalents. The Kolmogorov–Smirnov test was used to check for the normal distribution of quantitative data, provided here as the mean ± standard deviation (SD). Independent two-sample *t*-tests were used to compare data between the two groups. If the data were not normally distributed, the Wilcoxon rank-sum test was performed. One-way analysis of variance for repeated measures and paired *t*-tests were used to analyze the data from preoperative to postoperative examinations and between consecutive postoperative visits. The differences in data are reported with 95% confidence intervals (CIs). A two-tailed *P* value ≤0.05 was considered statistically significant for all analyses.

## 3. Results

Both the FS-LASIK Xtra and SMILE Xtra groups included 24 eyes of 12 patients, seven (58%) women and five (42%) men. The mean age was 25.25 ± 4.61 years and 25.08 ± 5.26 in the FS-LASIK Xtra group and SMILE group, respectively (*P*=0.935). There was no statistically significant difference in preoperative parameters between the groups (Table 1). In the FS-LASIK group, 79% (19/24) of eyes exhibited moderate myopia (spherical correction: −3.00∼−6.00D), and 21% (5/24) of eyes exhibited high myopia (spherical correction ≥ −6.00D). The corresponding proportions were 92% (22/24) and 8% (2/24) in the SMILE Xtra group.

### 3.1. Visual Acuity and Manifest Spherical Equivalent

**Figure 1:**
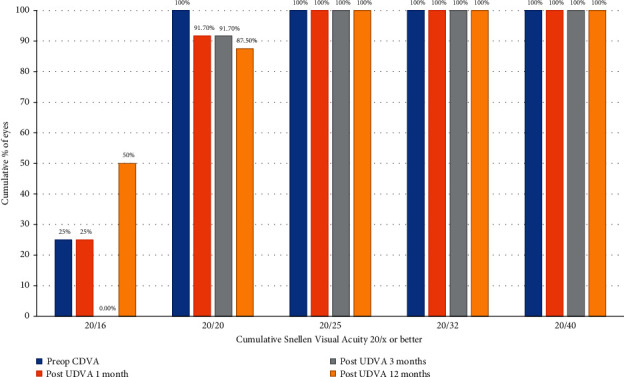
Cumulative Snellen visual acuity in the FS-LAISIK Xtra group at 1, 3, and 12 months.

**Figure 2:**
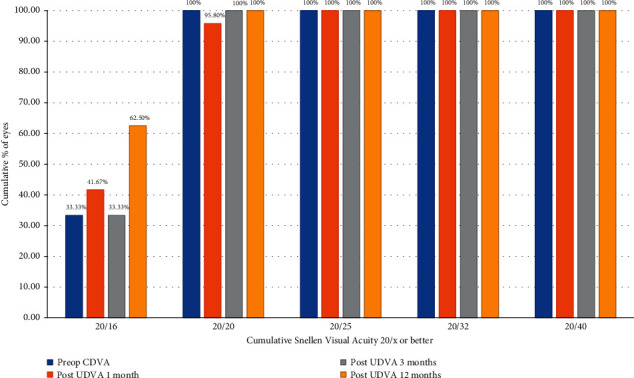
Cumulative Snellen visual acuity in the SMILE Xtra group at 1, 3, and 12 months.

The change in UDVA from pre- to postoperative was statistically significant in both groups after one day and 1, 3, and 12 months of surgery (*P* < 0.001), but not between groups (Tables 2–4). At 1, 3, and 12 months after surgery, a UDVA of 20/20 or better was achieved for 91.7%, 91.7%, and 87.5% of individuals, respectively, in the FS-LASIK group and 95.8%, 100%, and 100%, respectively, in the SMILE group (Figures 1 and 2).

**Figure 3:**
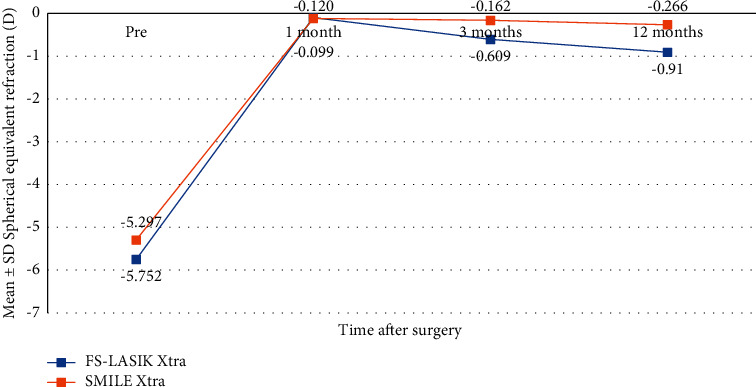
Manifest refractive spherical equivalent (MRSE) after FS-LASIK Xtra and SMILE Xtra at 1, 3, and 12 months.

The mean manifest spherical equivalent (MRSE) values were −0.099 ± 0.53, −0.609 ± 0.65, and −0.91 ± 0.17 at 1, 3, and 12 months after surgery, respectively, in the FS-LASIK group and −0.12 ± 0.47, −0.16 ± 0.37, and −0.05 ± 0.04, respectively, in the SMILE Xtra group (Figure 3). The change in mean MRSE from 3 months to 12 months was not significant in either group (*P*=0.899, 1.000) (Tables 2 and 3). However, the LASIK Xtra group had a significantly higher mean MRSE than the SMILE Xtra group at 3 and 12 months after surgery (*P*=0.006, 0.01) (Table 4). In the FS-LASIK Xtra group and SMILE Xtra groups, 79.2% and 87.5% of eyes were within ±0.50D of attempted correction at one month, and 87.5% and 100% of eyes were within ±1.00D of attempted correction at one month, respectively. At 12 months, 75% and 100% of eyes were within ±1.00D of attempted correction in the FS-LASIK Xtra and SMILE Xtra groups, respectively.

### 3.2. Keratometry

Changes in keratometry values after FS-LASIK Xtra and SMILE Xtra are shown in Tables 2 and 3. The *K*1 and *K*2 values increased at three months after surgery, but the change from 3 months to 12 months was not statistically significant in either group (FS-LASIK Xtra: *P*=0.082, 0.066; SMILE Xtra: *P*=0.086, 1.000). In addition, the *K*1 and *K*2 values at the one-month visit after SMILE Xtra were higher than those after FS-LASIK Xtra (*P*=0.024, 0.023). However, there were no significant differences after the 3-month and 12-month visits between the two groups (Table 4). There were no significant intragroup or intergroup differences in the mean surface regularity index (SRI) and surface asymmetry index (SAI) at each time point after surgery (Tables 2–4).

### 3.3. Ocular Response Analyzer Values

**Figure 4:**
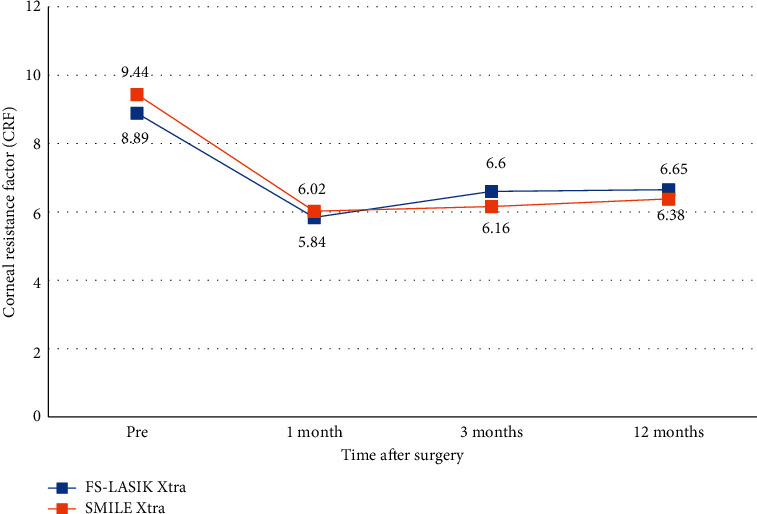
Corneal resistance factor (CRF) after FS-LASIK Xtra and SMILE Xtra at 1, 3, and 12 months.

**Figure 5:**
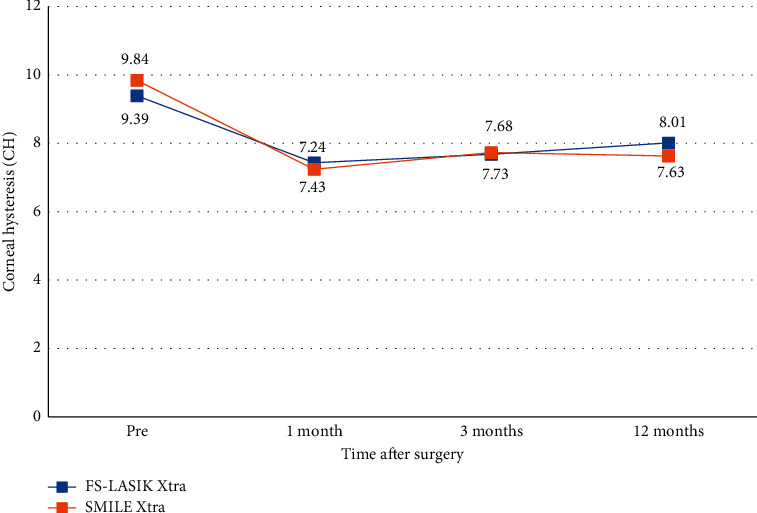
Corneal hysteresis (CH) after FS-LASIK Xtra and SMILE Xtra at 1, 3, and 12 months.

The FS-LASIK Xtra and SMILE Xtra groups showed a decline in CRF and CH at one month postoperatively and remained stable throughout the 12-month follow-up (Figures 4 and 5). The mean IOPcc of the FS-LASIK Xtra group was statistically higher than that of the SMILE Xtra group at three months (*P*=0.048), but there were no significant differences at 12 months postoperatively (Table 4). For CRF, CH, and IOPg values, there were no significant intragroup or intergroup differences at each time point after surgery (Tables 2–4).

### 3.4. Galilei Dual-Scheimpflug Analyzer Values

The CCT decreased significantly at one month postoperatively and started to increase over the next 12 months in both groups (*P* = 0.001) (Tables 2 and 3). There were no statistically significant differences between the groups at each time point after surgery (Table 4). Compared with the FS-LASIK Xtra group, the SMILE Xtra group had a higher mean SimK value at one month and higher posterior axial curvature values at three months postoperatively. However, the difference between preoperative and 12-month levels for both groups was not statistically significant (*P*=1.000) (Table 4). In terms of anterior instantaneous curvature values, there were no significant intergroup differences at each time point after surgery.

## 4. Discussion

Studies have shown that SMILE is a safe surgery with comparable outcome results to FS-LASIK [16–18]. However, there is still debate over whether SMILE is biomechanically stronger when compared with FS-LASIK. Because the collagen fibers in the anterior stroma are less disrupted following SMILE, the cornea should be stronger than after LASIK. Shetty et al. [19], in support of a preferential biomechanical recovery or healing response for SMILE, found that corneal deformation with higher forces returned to near preoperative levels by month six following SMILE, but not following LASIK. Theoretically, the cornea after SMILE Xtra might be biomechanically stronger than that after LASIK Xtra. At present, there is only one study to compare SMILE Xtra and FS-LASIK Xtra in their safety, efficacy, predictability, and stability, and it revealed that SMILE Xtra and FS-LASIK Xtra had similar one-year outcomes [15]. Nevertheless, the research did not measure the corneal biomechanical properties. In our study, we compared the visual acuity, refractive error, corneal morphology, and biomechanical properties between the two groups.

For the biomechanical properties, Konstantopoulos et al. performed an animal experimental study, and the result revealed that LASIK Xtra had significantly lower maximum posterior elevation (MPE) than LASIK and SMILE at week six. However, there was no significant MPE difference between the LASIK Xtra and SMILE Xtra groups at any visit points [14]. This suggests that taking CXL at the same time could lower the risk of ectasia after keratorefractive surgery due to the cornea's biomechanical stabilizing impact. Consistent with the previous study, we found that there were no significant intragroup or intergroup differences in the CRF, CH, and IOPcc values at each time point after surgery. Nevertheless, we should balance between biomechanical outcomes and other clinical outcomes, such as safety, efficacy, predictability, and stability. Thus, the proper prophylactic CXL should be performed.

Currently, there are no established prophylactic CXL regimens or patient selection criteria. Too much UVA energy could cause haze, but too little UVA energy would be insufficient to provide the required corneal strength. Some studies reported a different total UVA energy range from 0.8 to 5.4 J/cm^2^ [20–26], but the total UVA energy of 2.7 J/cm^2^ proved to be safe and well tolerated. Wu et al. [25] and Kohnen et al. [26] used a corneal cross-linking system at 30 mW/cm^2^ for 90 seconds (total energy: 2.7 J/cm^2^) to perform FS-LASIK Xtra. The results showed that FS-LASIK Xtra could effectively correct refractive error in patients with myopia with no significant complications during the 6-month and 12-month follow-up, indicating stability and morphologic changes similar to those with LASIK treatment. Liu et al. [15] also used a total irradiation UVA energy of 2.7 J/cm^2^ to perform SMILE Xtra; only three eyes showed mild haze, and it disappeared at the six-month visit. In our study, we also used the same protocol to perform prophylactic CXL. The patients not only had good clinical outcomes but also had no postoperative complications.

In terms of visual acuity, Kanellopoulos et al. [22, 27] found that 90.4% of eyes and 93.8% of eyes had a UDVA of 20/20 or better at one year and two years in the LASIK Xtra group. However, Lim et al. [28] proposed that 71.6% of eyes achieved a UDVA of 20/20 or better at one year of follow-up, 73.8% at two years of follow-up, and 65.1% at three years of follow-up. In our study, 87.5% of eyes achieved 20/20 or better at one year of follow-up. The controversy might be related to the different inclusion criteria of patients. Ganesh et al. [17] found that 95% of eyes exhibited a UDVA of 20/20 or better at one year after SMILE Xtra, and Osman et al. [29] proposed that 90% of eyes had a UDVA of 20/20 or better after two years in the SMILE Xtra group. In this study, 100% of eyes achieved 20/20 or better at one year of follow-up. Liu et al. [15] proposed that there were no statistical differences between FS-LASIK Xtra and SMILE Xtra. Consistent with the previous results, we also found no significant differences in the LogMAR UDVA between the two groups.

Regarding refractive error, Kanellopoulos et al. [22] found that LASIK Xtra had stability in MRSE correction at the 1-, 3-, 6-, 12-, and 24-month follow-up. In addition, Osman et al. [29] reported that SMILE Xtra had an improvement of MRSE at one month postoperatively and remained stable during 24 months of follow-up. In this study, we also found that the change in mean MRSE from 3 months to 12 months was not significant in either group. This result demonstrated that both procedures had good predictability. Tamayo [30] found that MRSE was 0.17 at one week and 0.49 at one month in the LASIK Xtra group. In contrast, we found a trend toward myopia regression in the LASIK Xtra group when compared with the SMILE Xtra group at 3 months and 12 months. This might be related to the fact that the LASIK Xtra group included a higher percentage of myopia patients and had a stronger flatting effect than the SMILE Xtra group.

*K*1 and *K*2 decreased significantly after the FS-LASIK Xtra and SMILE Xtra surgeries in our study. At the one-month follow-up, the *K*1 and *K*2 values were higher in the SMILE Xtra group than those in the FS-LASIK Xtra group, which might be related to the different flap or cap thickness between the two groups. However, the keratometry values remained stable from three months postoperatively in both groups. For corneal pachymetry, Kohnen et al. [26] found that corneal thickness was significantly higher after 12 months compared to one month after FS-LASIK Xtra. Furthermore, Osman et al. [29] proposed that SMILE Xtra exhibited a statistically significant decrease from one to three months, which increased again by six months and stabilized during the rest of the follow-up. Consistent with previous studies, we found that corneal thickness decreased significantly at one month postoperatively and started to increase over the next 12 months in both groups (*P*=0.001). The steady keratometry values and increased corneal thickness indicated that both surgical methods can maintain the stability of corneal morphology in the 12-month postoperative period.

To the best of our knowledge, this is the first study comparing the corneal biomechanics between the two procedures used for the correction of myopia and myopia astigmatism. Nevertheless, our study also has limitations. First, we did not examine the endothelial cell count because it has been proven that there was no significant effect in the endothelial cell count before and after FS-LASIK Xtra or SMILE Xtra. Second, the study needs a larger group and longer follow-up duration to observe regression and presence of ectasia because the ectasia appears after three years in most cases. The last limitation is the study's retrospective nature. Therefore, prospective and multicenter studies are needed to address this limitation.

## 5. Conclusions

In conclusion, FS-LASIK Xtra and SMILE Xtra are safe and effective in the correction of myopia and myopia astigmatism, and both procedures have the same effect on postoperative corneal morphology and biomechanics.

## Figures and Tables

**Table 1:** Demographics and preoperative data in the 2 groups.

	FS-LASIK Xtra	SMILE Xtra	*t*	*P* value
Age(years)	25.25 ± 4.61	25.08 ± 5.26	0.082	0.935
Female	7	7	—	—
Male	5	5	—	—
MRSE (diopters)	−5.752 ± 1.71	−5.297 ± 0.99	−1.131	0.265
UDVA (log MAR)	1.336 ± 0.27	1.313 ± 0.15	0.359	0.721
CDVA (log MAR)	−0.02 ± 0.04	−0.03 ± 0.04	0.624	0.535
CCT (*μ*m)	528.54 ± 20.52	531.58 ± 18.67	−0.537	0.594
*K*1 (diopters)	44.13 ± 1.57	44.65 ± 1.31	−1.25	0.218
*K*2 (diopters)	42.75 ± 1.52	43.38 ± 1.44	−1.485	0.144
K mean (diopters)	43.44 ± 1.51	44.00 ± 1.36	−1.348	0.184
SRI	0.16 ± 0.11	0.20 ± 0.18	−0.869	0.389
SAI	0.33 ± 0.11	0.29 ± 0.13	1.099	0.278
IOPcc	15.15 ± 2.29	15.15 ± 2.61	−0.006	0.995
IOPg	13.28 ± 2.60	13.83 ± 2.37	−0.772	0.444
CRF	8.89 ± 1.35	9.44 ± 1.23	−1.453	0.153
CH	9.39 ± 1.09	9.84 ± 1.30	−1.301	0.200
SimK mean	43.47 ± 1.46	44.05 ± 1.33	−1.417	0.163
Anterior instantaneous curvature	43.17 ± 1.52	43.80 ± 1.23	−1.561	0.125
Posterior axial curvature	−6.24 ± 0.25	−6.37 ± 0.23	1.764	0.084

MRSE = manifest refractive spherical equivalent; UDVA = uncorrected distance visual acuity; CDVA = corrected distance visual acuity; CCT = central corneal thickness; SRI = surface regularity index; SAI = surface asymmetry index; IOPcc = corneal compensated intraocular pressure; IOPg = Goldmann correlated intraocular pressure; CRF = corneal resistance factor; and CH = corneal hysteresis.

**Table 2:** The differences at 1, 3, and 12 months after FS-LASIK Xtra surgery.

	*UCVA*						*MRSE*					
	Preoperatively	1d		1 month	3 months		Preoperatively	1d		1 month	3 months	

1d	<0.001						0.003					
1 month	<0.001	0.322					0.001	1.000				
3 months	<0.001	0.776		1.000			0.003	0.754		0.011		
12 months	<0.001	1.000		1.000	1.000		0.002	0.331		0.007	0.899	

	*SimK mean*			*Anterior instantaneous curvature*			*Posterior axial curvature*			*CCT*		
	Preoperatively	1 month	3 months	Preoperatively	1 month	3 months	Preoperatively	1 month	3 months	Preoperatively	1 month	3 months
1 month	<0.001			<0.001			1.000			<0.001		
3 months	<0.001	0.088		<0.001	0.020		0.681	1.000		<0.001	0.427	
12 months	0.017	0.531	0.556	0.023	0.280	1.000	1.000	1.000	1.000	<0.001	0.007	0.001

	*K1*			*K2*			*SRI*			*SAI*		
	Preoperatively	1 month	3 months	Preoperatively	1 month	3 months	Preoperatively	1 month	3 months	Preoperatively	1 month	3 months
1 month	<0.001			<0.001			1.000			0.379		
3 months	<0.001	<0.001		<0.001	0.028		0.162	1.000		0.742	1.000	
12 months	0.001	0.012	0.082	<0.001	0.021	0.066	1.000	1.000	0.529	1.000	1.000	1.000

	*IOPcc*			*IOPg*			*CRF*			*CH*		
	Preoperatively	1 month	3 months	Preoperatively	1 month	3 months	Preoperatively	1 month	3 months	Preoperatively	1 month	3 months
1 month	0.144			<0.001			0.002			0.042		
3 months	0.708	1.000		0.016	1.000		0.027	1.000		0.448	0.489	
12 months	1.000	1.000	0.464	0.324	0.229	0.379	0.229	0.107	0.332	0.998	0.345	1.000

UDVA = uncorrected distance visual acuity; MRSE = manifest refractive spherical equivalent; CCT = central corneal thickness; SRI = surface regularity index; SAI = surface asymmetry index; IOPcc = corneal compensated intraocular pressure; IOPg = Goldmann correlated intraocular pressure; CRF = corneal resistance factor; CH = corneal hysteresis.

**Table 3:** The differences at 1, 3, and 12 months after SMILE Xtra surgery.

	*UCVA*						*MRSE*					
	Preoperatively	1d		1 month	3 months		Preoperatively	1d		1 month	3 months	

1d	<0.001						<0.001					
1 month	<0.001	0.039					<0.001	0.001				
3 months	<0.001	0.059		1.000			<0.001	0.003		0.094		
12 months	<0.001	0.043		1.000	1.000		<0.001	0.314		0.331	1.000	

	*SimK mean*			*Anterior instantaneous curvature*			*Posterior axial curvature*			*CCT*		
	Preoperatively	1 month	3 months	Preoperatively	1 month	3 months	Preoperatively	1 month	3 months	Preoperatively	1 month	3 months
1 month	<0.001			<0.001			1.000			<0.001		
3 months	<0.001	0.002		<0.001	0.048		0.385	0.518		<0.001	0.306	
12 months	<0.001	0.001	1.000	<0.001	<0.001	1.000	0.044	1.000	1.000	<0.001	<0.001	<0.001

	*K1*			*K2*			*SRI*			*SAI*		
	Preoperatively	1 month	3months	Preoperatively	1 month	3 months	Preoperatively	1 month	3 months	Preoperatively	1 month	3 months
1 month	<0.001			<0.001			0.548			0.830		
3 months	<0.001	0.025		<0.001	0.001		1.000	1.000		0.611	1.000	
12 months	<0.001	0.002	0.858	<0.001	0.001	1.000	1.000	0.562	1.000	0.217	1.000	1.000

	*IOPcc*			*IOPg*			*CRF*			*CH*		
	Preoperatively	1 month	3months	Preoperatively	1 month	3 months	Preoperatively	1 month	3 months	Preoperatively	1 month	3 months
1 month	0.073			0.001			0.001			0.058		
3 months	1.000	0.745		0.049	1.000		0.009	1.000		0.138	1.000	
12 months	0.156	0.953	1.000	0.003	1.000	0.663	0.021	1.000	0.873	0.220	1.000	1.000

UDVA = uncorrected distance visual acuity; MRSE = manifest refractive spherical equivalent; CCT = central corneal thickness; SRI = surface regularity index; SAI = surface asymmetry index; IOPcc = corneal compensated intraocular pressure; IOPg = Goldmann correlated intraocular pressure; CRF = corneal resistance factor; CH = corneal hysteresis.

**Table 4:** Postoperative data of FS-LASIK Xtra and SMILE Xtra groups.

	1 month			3 months			12 months		
	FS-LASIK Xtra	SMILE Xtra	*P* value	FS-LASIK Xtra	SMILE Xtra	*P* value	FS-LASIK Xtra	SMILE Xtra	*P* value
MRSE (diopters)	−0.099 ± 0.53	−0.120 ± 0.467	0.886	−0.609 ± 0.65	−0.162 ± 0.37	0.006^*∗*^	−0.91 ± 0.17	−0.266 ± 0.52	0.01^*∗*^
UDVA (log MAR)	−0.014 ± 0.04	−0.033 ± 0.06	0.192	−0.010 ± 0.049	−0.023 ± 0.04	0.344	0.012 ± 0.06	−0.050 ± 0.04	0.186
CCT (*μ*m)	429.25 ± 29.04	432.46 ± 24.69	0.682	436.88 ± 34.45	431.71 ± 26.26	0.562	461.5 ± 15.11	460.75 ± 8.96	0.906
*K*1 (diopters)	38.86 ± 1.52	39.95 ± 1.73	0.024^*∗*^	39.50 ± 1.55	40.18 ± 1.92	0.179	40.14 ± 0.99	41.33 ± 2.75	0.279
*K*2 (diopters)	38.07 ± 1.47	39.20 ± 1.84	0.023^*∗*^	38.65 ± 1.43	39.43 ± 1.93	0.115	39.06 ± 0.94	40.55 ± 2.98	0.215
SRI	0.32 ± 0.26	0.24 ± 0.19	0.233	0.29 ± 0.20	0.24 ± 0.15	0.324	0.19 ± 0.15	0.18 ± 0.21	0.881
SAI	0.67 ± 0.40	0.57 ± 0.45	0.415	0.57 ± 0.29	0.51 ± 0.17	0.408	0.45 ± 0.27	0.60 ± 0.17	0.196
IOPcc	13.35 ± 2.09	13.20 ± 2.22	0.800	14.33 ± 2.01	13.20 ± 1.82	0.048^*∗*^	13.31 ± 1.26	13.81 ± 1.12	0.416
IOPg	8.75 ± 2.37	8.75 ± 2.19	0.995	10.01 ± 2.28	8.90 ± 1.51	0.053	9.36 ± 2.46	8.64 ± 0.33	0.436
CRF	5.84 ± 1.21	6.02 ± 0.96	0.555	6.60 ± 1.30	6.16 ± 0.61	0.143	6.65 ± 1.64	6.38 ± 0.82	0.806
CH	7.43 ± 0.98	7.24 ± 1.65	0.641	7.68 ± 0.87	7.73 ± 0.78	0.849	8.01 ± 1.71	7.63 ± 0.74	0.442
SimK mean	37.85 ± 1.55	39.27 ± 1.90	0.007^*∗*^	38.52 ± 1.64	39.52 ± 2.05	0.068	39.52 ± 1.94	40.67 ± 2.93	0.371
Anterior instantaneous curvature	39.70 ± 1.38	39.99 ± 1.67	0.505	40.13 ± 1.45	40.21 ± 1.85	0.881	40.94 ± 1.61	41.23 ± 2.56	0.793
Posterior axial curvature	−6.54 ± 0.54	−6.59 ± 0.24	0.643	−6.44 ± 0.31	−6.61 ± 0.24	0.031^*∗*^	−6.39 ± 0.49	−6.63 ± 0.33	0.281

MRSE = manifest refractive spherical equivalent; UDVA = uncorrected distance visual acuity; CCT = central corneal thickness; SRI = surface regularity index; SAI = surface asymmetry index; IOPcc = corneal compensated intraocular pressure; IOPg = Goldmann correlated intraocular pressure; CRF = corneal resistance factor; CH = corneal hysteresis. ^*∗*^*P* < 0.05.

## Data Availability

The datasets obtained and/or analyzed during the current study are available from the corresponding author on reasonable request.
